# Antiphospholipid Antibody Testing: An Audit on Testing Practices in a Public Tertiary Care Center

**DOI:** 10.3390/jcm13010243

**Published:** 2023-12-31

**Authors:** Eman M. Mansory, Hatem M. Alahwal, Salem M. Bahashwan, Osman Radhwi, Abdullah T. Almohammadi, Yassir Daghistani, Jamil Al-Mughales, Ahmed S. Barefah

**Affiliations:** 1 Hematology Department, Faculty of Medicine, King Abdulaziz University, Jeddah 21589, Saudi Arabia; halahwal@kau.edu.sa (H.M.A.); smbahashwan1@kau.edu.sa (S.M.B.); oradhwi@kau.edu.sa (O.R.); atalmohammade@kau.edu.sa (A.T.A.); asbarefah@kau.edu.sa (A.S.B.); 2 Hematology Research Unit, King Fahd Medical Research Center, King Abdulaziz University, Jeddah 21589, Saudi Arabia; 3 Department of Medicine, College of Medicine, University of Jeddah, Jeddah 23890, Saudi Arabia; ydaghistani@uj.edu.sa; 4 Department of Clinical Laboratories, Diagnostic Immunology Division, King Abdulaziz University, Jeddah 21589, Saudi Arabia; jmughales@kau.edu.sa; 5 Department of Clinical Microbiology and Immunology, King Abdulaziz University, Jeddah 21589, Saudi Arabia

**Keywords:** antiphospholipid antibody, APS, testing practice

## Abstract

Background: Antiphospholipid antibodies (aPLs) are antibodies directed against cell membrane components and can be associated with clinical features or be asymptomatic. Testing and interpreting these antibodies is associated with many challenges and pitfalls in clinical practice. Objective: To review all antiphospholipid antibody testing and describe the testing practices, indications for testing and interpretation of results to infer local challenges with aPL testing and subsequently address ways to overcome those challenges. Methods: This is a retrospective analysis of all aPL testing done in a tertiary center between 2014 and 2018. Characteristics of study patients collected through chart review were described using the mean and standard deviation for continuous variables and proportion for categorical variables. Group differences were compared between patients with any aPL-positive result and those with no positive result using chi-square or Fisher’s exact test as appropriate for categorical variables and a simple regression model for numerical variables. Results: Among 414 patients undergoing aPL testing, mainly adult females, 62 (14.9%) patients had at least one positive antibody, of those, 26 (42%) had repeat testing done. Testing was mostly done for obstetric indication (107, 25.8%), with 36 patients having one or two early pregnancy losses <10 weeks as their testing indication. A total of 27 (6.5%) patients were labeled with APS/possible APS based on chart review, but on review of the testing of those patients according to classification criteria, only nine patients satisfied the criteria for APS. Conclusion: This study highlights the clinical challenges associated with aPL testing, including the controversies around indication for testing, the low rates of repeat testing to confirm persistence, and the common misinterpretation of results. Having an aPL testing profile, explicit reference ranges, results commentary, and close interaction between ordering physicians and laboratory staff might be starting points to overcome these challenges.

## 1. Introduction

Antiphospholipid syndrome (APS) is an autoimmune thrombogenic disorder characterized by the presence of persistent antiphospholipid antibodies (aPL) in addition to the presence of clinical manifestations, whether venous or arterial thrombosis or pregnancy morbidities [1]. Given the different presentations APS can manifest with, testing tends to be shared between many specialties, including hematology, cardiology, neurology, rheumatology, vascular, and obstetrics, in addition to general practice and family physicians. These different specialties may carry their own inherent biases when it comes to testing practices. However, the importance of correctly diagnosing APS cannot be overemphasized since it determines many subsequent diagnostic and management decisions including the potential need of lifelong anticoagulation with its associated risks [2].

Antiphospholipid antibodies are a heterogeneous group of autoantibodies that are directed against phospholipid-binding proteins. Those antibodies are detected using different assays that have varying degrees of sensitivity and specificity. The ones that are currently included in the classification criteria are lupus anticoagulant (LAC), anticardiolipin antibodies (aCL) IgG and IgM, and anti-B2-glycoprotein antibodies (aB2GP) IgG and IgM [3]. The testing and interpretation of these antibodies is associated with many challenges and pitfalls in clinical practice, including interference with medications, the need for repeat testing to establish persistence, the overuse of testing in specific clinical scenarios that are not necessarily evidence-based, and the misinterpretation of reported values given the different cutoffs used due to interlaboratory variations and a lack of international standards [2,4]. Current guidance does not consider weakly positive levels of aCL and aB2GP antibodies to be clinically relevant and a level at or above the 99th percentile is needed to establish positivity [3]. Although there are many studies describing the challenges around aPL testing and elaborating on best testing procedures, available assays and their shortcomings, and summarizing current consensus guidelines [5,6,7,8], to our surprise, there were not as much studies that have tried to quantify the effect of these challenges on clinical practice and patient diagnosis accuracy. An audit done by Favaloro et al. identified a wide variety of clinical ordering backgrounds and found that 27% of orders were for obstetric patients with no positive results among those patients, suggesting a need to review local testing guidelines [9].

Recently, the American College of Rheumatology (ACR) and the European League Against Rheumatism (EULAR) updated the antiphospholipid syndrome classification criteria [3]. These criteria were mainly developed to provide a better definition for patients involved in research studies rather than for clinical practice. The new criteria carry a specificity of 99% versus 86% for the old ones, and a sensitivity of 84% versus 99%. Moreover, they are more complex than the previously used revised Sapporo criteria [10]. However, the 2023 ACR/EULAR APS classification criteria will likely be of benefit in guiding the evaluation of aPL-positive patients, specifically in trying to avoid the overdiagnosis of APS which is a common clinical practice issue. When compared to the Sapporo criteria, there are significant changes to the clinical aspect of the guidelines which now include six clinical domains (venous thromboembolism, arterial thrombosis, microvascular disease, obstetric, cardiac valve and hematology). On the other hand, there is little change in the laboratory aspect of the criteria.

In this study, we will review the aPL testing done in our tertiary care center to recognize the practice pattern, characteristics of patients undergoing testing, and testing indications and interpretation to identify local issues in aPL testing and areas amenable to improvement.

## 2. Methods

This is a retrospective quality improvement audit of all antiphospholipid antibody testing done in Kind Abdulaziz University Hospital, a public tertiary care center in Jeddah, Saudi Arabia. All aPL tests conducted between the years 2014 and 2018 were retrieved (LAC, aCL IgG and IgM, and aB2GP IgG and IgM) in addition to a chart review to record patient demographics (age, sex, weight, and height), preliminary and final patient diagnosis and comorbidities present, anticoagulation used, detailed thrombosis history, including venous events (pulmonary embolism, deep vein thrombosis, or cerebral vein thrombosis) or arterial events (stroke, myocardial infarction (MI), or limb ischemia), as well as obstetric history (miscarriage, premature labor, pre-eclampsia, and eclampsia) and other laboratory investigations including prothrombin time (PT), partial thromboplastin time (PTT), platelet count, and anti-nuclear antibody (ANA) and anti-double stranded DNA antibody (DsDNA) testing.

The study included all aPL testing done regardless of indication. Patients had to be adults with any one of the aPL antibodies tested, whether inpatients or outpatients, in the period between January 2014 and December 2018. The study includes testing done at our center only and results obtained from outside centers were excluded to avoid confusion by the different laboratory assays used at different laboratories. Lupus anticoagulant testing included in the sample required individuals to be off anticoagulants to be accepted in the lab. This study was approved as a quality assurance project by the institution’s Unit of Biomedical Ethics, Research Ethics Committee, NCBE registration number (HA-02-J-008) Reference No. 46/22, obtained on 2 March 2022. 

### 2.1. Assays and Test Procedures

LAC testing was performed using dilute Russell’s viper venom time (dRVVT) Werfen solution on ACL TOP 50 instrumentation. It was considered positive when above the 99th percentile of the distribution; at our lab, this is equivalent to a ratio is higher than 1.3 [11]. Assessment of ACA IgG and IgM and aB2GP IgG and IgM in our facility was performed by ELISA using a commercial kit (Alegria; ORGENTEC Diagnostika GmbH, Mainz, Germany). The test was considered low positive if it was between 20 and 40 U/mL and significantly positive if >40 U/mL which met the 99th percentile [10,12].

### 2.2. Analysis Plan

This study is mainly descriptive. Characteristics of study patients who met inclusion criteria were described using the mean and standard deviation for continuous variables and proportion for categorical variables. Group differences were compared between patients with any aPL-positive results and those with no positive results using chi-square or Fisher’s exact test as appropriate for categorical variables and a simple regression model for numerical variables. All statistical analyses were performed using a standard software package (StataCorp. 2023. Stata Statistical Software: Release 18). 

## 3. Results

A total of 414 patients underwent testing for antiphospholipid antibodies during the analysis period. Interestingly, a great majority were females (85%) and 366 (88.4%) were adult patients. The characteristic of the patients including their age, BMI, indication for testing based on chart review, comorbidities present and as well as history of venous thromboembolism (VTE), arterial events, or pregnancy mortality events is presented in Table 1. The results are presented grouping patients into those who had any positive aPL tests vs. those with no positive tests. Characteristics that were significantly prominent in patients with aPL positivity were a history of heart failure, history of autoimmune disease, prolonged aPTT, and the presence of positive ANA or DsDNA titers. Overall, 144 patients had repeat testing done whether it was for confirming a positive result or for repeat testing due to a separate presentation.

Testing was mostly done for obstetric indication (107, 25.8%), as part of VTE workup (76, 18.4%), as part of the workup of lupus or autoimmune disease (48, 11.6%), and arterial events (22, 5.3%). In the remaining 161 (38.8%) patients, the reason for sending for aPL testing was not entirely clear based on chart review. There was no statistically significant difference in the proportion of patients with VTE, arterial disease, early pregnancy losses, or pregnancy complications (premature labor, eclampsia, and pre-eclampsia) between patients with all negative results and patients with any positive results. Among the testing done for obstetric indications, 14 patients had pregnancy complications and 93 patients had a history of miscarriage (36 patients had one or two miscarriages < 10 weeks, 47 patients had > or = 3 recurrent early miscarriage). 

Only 79 (19.1%) patients had the full set of antibodies tested ordered at once, while the remaining patients had at least one test missing. The least requested antibody subtype was aB2GP, both IgG and IgM. The breakdown of the number of each antibody tested and results of testing are summarized in Table 2. 

Looking at the first set of aPL antibodies, 62 (14.9%) patients had at least one positive test in initial testing (whether the test was mildly or significantly positive), only 26 of those had repeat testing ordered. In patients who had a repeat set of testing, the average mean duration between testing was 537 days (range 5,2624). We also noticed gaps in testing that were related to the absence of reagents.

Review of the patients’ charts revealed that 27 (6.5%) patients were labeled with APS/possible APS, but upon review of the testing of those patients according to classification criteria, only nine patients satisfied the criteria for APS. Of the 27 patients labeled as APS, six patients were diagnosed with secondary APS related to systematic lupus erythematosus (SLE). There was a total of 49 patients with SLE in the sample and so the rate of APS in patients with SLE came to 12.2%. 

**Figure 1:**
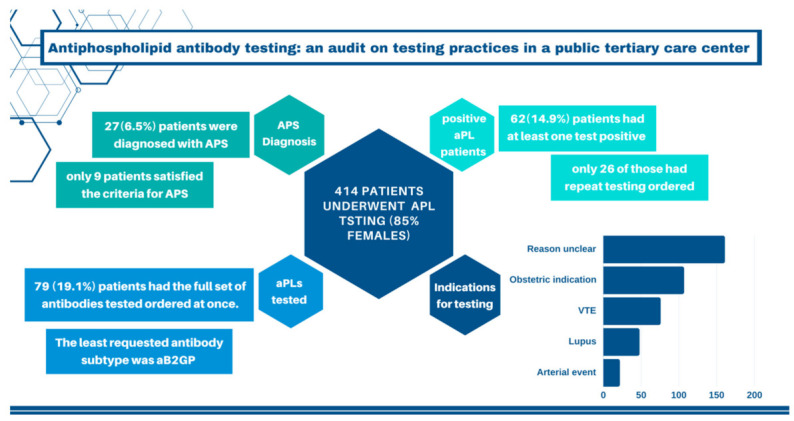
Summary of study results.

With regards to anticoagulation, 113 (27.3%) patients were on anticoagulants as follows 58 patients on warfarin, 28 patients on therapeutic dose low molecular weight heparin (LMWH), 20 patients on therapeutic dose unfractionated heparin (UFH), and 7 patients on a direct oral anticoagulant (DOAC). Unfortunately, the exact indication for the anticoagulation was not collected. Patients were considered to be “on anticoagulation even if it was for a very short period of time”. Also, patients were switched between different agents frequently and so patients who were on an oral agent long term were counted as on warfarin/DOAC, even if this was interrupted or replaced by UFH or LMWH at various doses during admission for example. See Figure 1 for a visual summary of the study results.

## 4. Discussion

In this paper, we review 414 patients who had undergone antiphospholipid antibody testing at least once at a tertiary care center between 2014 and 2018. The sample was dominated by females, and 25.8% of testing was related to obstetric reasons; adjudication of these reasons revealed that 34% of these patients did not satisfy clinical criteria suggesting APS. We also noted that only 19% of the patient had the full set sent at once, and in the remaining patients, it was fragmented on more than one test order, with discrepancy in what subtypes of antibodies were tested in different patients. LAC was the most frequently requested antibody, followed by aCL testing, and aB2GP testing. Among patients with a positive test result, only about half had repeat testing done and only 33% of the patients labeled with an APS diagnosis satisfied the laboratory criteria for APS.

This audit highlights many important points that are commonly encountered in clinical practice around antiphospholipid testing; first is the controversy around the timing for testing for aPL antibodies in obstetric practice in patients with early pregnancy loss [13]. Currently, the American college of obstetricians and gynecologist (ACOG) and the royal college of obstetricians and gynecologists (RCOG) suggest considering aPL testing after two or more and three or more early pregnancy losses, respectively [14,15]. Testing after one event likely carries a very low yield as only 10–15% of miscarriages are related to APS [16,17]. In addition, this practice has the potential to initiate anticoagulant prophylaxis or aspirin unnecessarily in this patient group. Another aPL testing review from a tertiary center in Australia found a similar dominance of obstetric indications for testing including abortions, stillbirth, and infertility, accounting for 27% of tests orders [9]. This raises the question whether more refined testing indications should be specifically described in this patient population.

The interpretation of aPL is complex in clinical practice due to many factors, including the absence of assay standardization [4], the use of different cutoffs of a positive test across laboratories, and the challenges related to reading results of LAC given the requirement of multiple steps and the many pre-, post-, and analytical factors at play [6,18,19]. Several reports have recommended an interpretive comment in electronic health records in addition to a close interaction between clinicians and pathologists to enhance the diagnostic process and ensure the correct labeling of patients [7,12]. After this audit, we are in hoping to implement these changes at our institution, namely, labeling the aCL and aB2GP levels that are consistent with criteria and adding an interpretive comment attached to the test report in the electronic health record (HER) to avoid confusion.

Moreover, the discrepancy in what antibody subtypes were tested in different patients and the low percentage (19%) of patients who had undergone full aPL workup suggest that an aPL ordering module/panel that facilitates the collective ordering of all three assays (LAC, aCL, and aB2GP) on the same sample would be helpful. This would increase the diagnostic utility and lead to better patient risk assessment and management, rather than entering five different orders and having fragmented results. This has also been advised in the recent guidance by the International Society on Thrombosis and Haemostasis Scientific and Standardization Subcommittee (ISTH-SSC) for lupus anticoagulant/antiphospholipid antibodies [12,20].

Between testing done too soon, and testing done many months later, the study highlights the challenges of repeating testing in a timely manner, whether related to patients’ lack of follow up, missed repeat testing after patient discharge, interfering medications requiring delayed testing to establish accurate results, and other factors. A study done in Korea reported that less than 10% of patients with initial positive tests had follow-up tests at intervals of more than 12 weeks. It also found that patients with persistently positive antibodies at an interval of more than 12 weeks had lower clinical symptoms than patients who had repeat testing between 6 and 12 weeks [21]. Ways to address factors leading to low follow-up testing and methods to facilitate repeat testing to be at least 12 weeks apart need further analysis. We also note that aB2GP antibodies were less frequently tested as compared to aCL and LAC. We wonder if this was related to the traditional focus on aCL and LAC testing before B2GP was added to the modified Sapporo criteria in 2006 as an independent risk factor for thrombosis and pregnancy [10]. In about 10% of APS patients, aB2GP may be the only positive antibody and so testing for it is of significance but might be hindered by cost, availability, and technical barriers.

The APS classification criteria’s main goal is to provide a highly specific tool to diagnose patients for research purposes; this translates in some patients with APS who do not satisfy criteria in clinical practice, and in this study, 33% who were labeled as APS patients did not satisfy criteria. This is not too uncommon as previous studies have reported that up to 68% of patients diagnosed with APS did not meet the laboratory criteria, whether due to incorrectly labeling lower titer for antibodies as positive, repeat testing not done, or done too soon [22,23].

There was no statistical difference in the rates of VTE events, arterial events, or rates of pregnancy complications between patients with positive aPLs and those with negative testing. This is not surprising and is a reminder to keep in mind that both thrombosis events and pregnancy complications are frequent and are related to several etiologies unrelated to aPL [24]. Among patients with a first unprovoked VTE event, only about 9% of patients were diagnosed with APS [25], and about 10-11% of patients with an MI who were younger than 75 were found to have positive aCL or aB2GP [26]. A meta-analysis that included 5217 young patients with cardiovascular events found that 17% of patients had positive aPL [27].

Patients with SLE are known to be at a higher risk of APS than the general population [28]. In the general population, 1–5% of normal individuals may have a positive aPL [29]. However, this goes up to 40% in patients with SLE but only about 10–15% have a clinical diagnosis of APS [30]. This was reflected in our study, which found that 12% of patients with SLE captured in the study had APS, and that there was statistically significantly more ANA and ds-DNA positivity in patients with any positive aPL. It has even been reported that patients with primary APS with no associated SLE have circulating ANA and dsDNA [31]. Collectively, these observations highlight the importance of screening for APS in patients with SLE as recommended by recent guidelines [32].

APS is a complex disorder and our understanding of its diagnosis and management is continuously evolving. Moreover, the decisions around patient care are influenced by many factors including the patient’s history, clinical presentation, and many other variables in addition to the laboratory results, and the results of this study should be interpreted in that context. With the development of many other non-criteria antiphospholipid antibodies that are currently available (antidomain I B2GPI IgG antibodies, IgA aCL and aB2GPI, antiphosphatidylserine/prothrombin antibodies (aPS/PT), and anti-annexin A5 antibodies), it is likely that the challenges around antiphospholipid testing and standardization are only getting more complicated. Currently, routine testing for non-criteria antibodies is not recommended as the evidence so far suggests an overlap between these antibodies and the current criteria-defined antibodies [33,34], but emerging data are suggesting an added value of non-criteria aPLs in improving the diagnostic accuracy in APS [35,36], and a role in risk stratification, especially for aPS/PT antibodies [18]. Finally, a possible significant value is described in patients with “seronegative APS” with an increase in the antibody positivity rate from 65.7% when testing for criteria aPLs only to 87.4% when non-criteria aPLs were tested [35].

Finally, this study focusses the attention on real-world issues associated with ordering and interpreting aPL tests in clinical practice and points to a need for continuous educational efforts on good vs. bad testing practices. It also calls for simple system changes that may go a long way, such as creating aPL testing modules in HER, adding criteria defined reference ranges, as well as providing an interpretive comment. This study has several limitations, including the possibility of repeat testing outside the center, the limited insight into the motivations behind testing given the retrospective nature of the study, and the barriers related to data collection from HER. On the other hand, we believe it is fairly representative of testing practices and issues associated with antiphospholipid testing that are observed in everyday clinical life. This study is a call for further studies to unify testing indications, assays, and reference ranges, as well as to address ways to ensure appropriate repeat testing and interpretation to improve the diagnostic accuracy of a complicated and rapidly evolving clinical diagnosis. 

## 5. Conclusions

Antiphospholipid syndrome is a complex diagnosis due to variable presenting symptoms, multiple laboratory testing requirements, and the combination of both clinical and laboratory criteria to reach an accurate diagnosis. The testing for antiphospholipid antibodies at our center was significantly dominated by testing for an obstetric indication. A limited number of patients had complete antibody testing done at once with discrepancies in what subtypes of antibodies were tested in different patients; aB2GP antibodies were less frequently tested as compared to aCL and LAC. Among patients with a positive test result, only about half had repeat testing done and in patients who were labeled with APS, one third had satisfied the full criteria of APS.

The study highlights the importance of having an aPL testing profile to ensure complete testing, clarification of reference ranges, a commentary attached to the results, and a close interaction between ordering physicians and laboratory staff to ensure the best diagnostic accuracy of this often challenging and developing condition.

## Figures and Tables

**Table 1:** Characteristics of all patients who had antiphospholipid antibody testing done grouped by test results.

	Any Positive Antibody Test			
	Negative	Positive	Total	Test
N	352 (85%)	62 (15%)	414 (100%)	
**Demographics**				
Male	48 (14%)	15 (24%)	63 (15%)	0.033
BMI—mean (SD)	29 (16)	27 (7.5)	29 (15)	0.32
Age—mean (SD)	38 (14)	36 (14)	38 (14)	0.39
**Comorbidities**				
Heart failure	13 (3.7%)	7 (11%)	20 (4.8%)	0.01
Hypertension	46 (13%)	12 (19%)	58 (14%)	0.19
Active malignancy	3 (0.85%)	1 (1.6%)	4 (0.97%)	0.57
Respiratory disease	12 (3.4%)	2 (3.2%)	14 (3.4%)	0.94
Diabetes mellitus	36 (10%)	5 (8.1%)	41 (9.9%)	0.6
CKD	10 (2.8%)	5 (8.1%)	15 (3.6%)	0.042
Anemia	19 (5.4%)	3 (4.8%)	22 (5.3%)	0.86
Autoimmune disorder	56 (16%)	17 (27%)	73 (18%)	0.028
Neurological disorder	11 (3.1%)	3 (4.8%)	14 (3.4%)	0.49
**Events**				
Arterial thrombosis	29 (8.2%)	5 (8.1%)	34 (8.2%)	0.96
History of VTE	65 (18%)	10 (16%)	75 (18%)	0.66
Pregnancy complications *^+^	10 (3.3%)	4 (8.5%)	14 (4%)	0.089
History of early pregnancy loss *	78 (26%)	8 (17%)	86 (25%)	0.2
**Laboratory tests**				
Positive ANA	161 (46%)	42 (68%)	203 (49%)	0.0014
Positive ds-DNA	24 (6.8%)	14 (23%)	38 (9.2%)	<0.001
aPTT—mean (SD)	34 (13)	40 (17)	35 (14)	0.0053
Platelets count—mean (SD)	281 (141)	268 (115)	279 (137)	0.54

BMI = body mass index, SD = standard deviation, CKD = chronic kidney disease, VTE = venous thromboembolism, ANA = anti-nuclear antibody, ds-DNA = anti-double stranded DNA, aPTT = activated partial thromboplastin time. * Pregnancy complications and history of early pregnancy loss numbers are presented for female patients only. ^+^ Pregnancy complications include: premature labor, placental abruption, pre-eclampsia, and eclampsia. Note: using Pearson’s test across levels of any positive result for categorical variables, and using test regress across levels of any positive result for numerical variables (BMI, age, aPTT, and platelets count).

**Table 2:** The frequency and results of the different antiphospholipid antibody subtypes.

First Set of Antibody Tests	Test Results n (%)		Second Set of Antibody Tests	Test Results n (%)	
Anticardiolipin antibody IgG	Negative Low positive High positive Not done	214 (51.7%) 4 (1.0%) 5 (1.2%) 191 (46.1%)	Anticardiolipin antibody IgG	Negative Low positive High positive Not done	77 (18.6%) 7 (1.7%) 1 (0.2%) 329 (79.5%)
Anticardiolipin antibody IgM	Negative Low positive High positive Not done	301 (72.7%) 10 (2.4%) 4 (1.0%) 99 (23.9%)	Anticardiolipin antibody IgM	Negative High positive Not done	97 (23.4%) 3 (0.7%) 314 (75.8%)
Anti-glycoprotein antibody IgG	Negative Low positive High positive Not done	136 (32.9%) 1 (0.2%) 5 (1.2%) 272 (65.7%)	Anti-glycoprotein antibody IgG	Negative Low positive High positive Not done	58 (14.0%) 1 (0.2%) 3 (0.7%) 352 (85.0%)
Anti-glycoprotein antibody IgM	Negative Low positive High positive Not done	186 (44.9%) 2 (0.5%) 2 (0.5%) 224 (54.1%)	Anti-glycoprotein antibody IgM	Negative High positive Not done	72 (17.4%) 2 (0.5%) 340 (82.1%)
Lupus anticoagulant	Negative Positive Not done	229 (55.3%) 40 (9.7%) 146 (35.3%)	Lupus anticoagulant	Negative Positive Not done	67 (16.2%) 16 (3.1%) 331 (80.0%)

## Data Availability

The required data were obtained from the hospital information system. The data can be obtained by contacting the corresponding author and are subject to the rules and regulations of ethical standards.
